# Enhancement of ecosystem carbon uptake in a dry shrubland under moderate warming: The role of nitrogen‐driven changes in plant morphology

**DOI:** 10.1111/gcb.15823

**Published:** 2021-08-16

**Authors:** Dario Liberati, Gabriele Guidolotti, Giovanbattista de Dato, Paolo De Angelis

**Affiliations:** ^1^ Department for Innovation in Biological, Agro‐Food and Forest Systems (DIBAF) University of Tuscia Viterbo Italy; ^2^ Present address: Institute of Research on Terrestrial Ecosystems (IRET) National Research Council (CNR) Porano TR Italy; ^3^ Present address: Council for Agricultural Research and Economics (CREA) – Research Centre for Forestry and Wood Arezzo Italy

**Keywords:** canopy photosynthesis, *Cistus monspeliensis*, dry shrubland, experimental warming, net ecosystem exchange, nutrient limitations, shoot size, temperature limitations to plant growth

## Abstract

Net ecosystem CO_2_ exchange is the result of net carbon uptake by plant photosynthesis and carbon loss by soil and plant respiration. Temperature increases due to climate change can modify the equilibrium between these fluxes and trigger ecosystem‐climate feedbacks that can accelerate climate warming. As these dynamics have not been well studied in dry shrublands, we subjected a Mediterranean shrubland to a 10‐year night‐time temperature manipulation experiment that analyzed ecosystem carbon fluxes associated with dominant shrub species, together with several plant parameters related to leaf photosynthesis, leaf morphology, and canopy structure. Under moderate night‐time warming (+0.9°C minimum daily temperature, no significant reduction in soil moisture), *Cistus monspeliensis* formed shoots with more leaves that were relatively larger and denser canopies that supported higher plant‐level photosynthesis rates. Given that ecosystem respiration was not affected, this change in canopy morphology led to a significant enhancement in net ecosystem exchange (+47% at midday). The observed changes in shoot and canopy morphology were attributed to the improved nutritional state of the warmed plants, primarily due to changes in nitrogen cycling and higher nitrogen resorption efficiency in senescent leaves. Our results show that modifications in plant morphology triggered by moderate warming affected ecosystem CO_2_ fluxes, providing the first evidence for enhanced daytime carbon uptake in a dry shrubland ecosystem under experimental warming.

## Graphical abstract

Temperature increases due to climate change can modify the equilibrium between net carbon uptake by plant photosynthesis and carbon loss by soil and plant respiration. We subjected a dry shrubland to a 10‐year night‐time temperature manipulation experiment (+0.9°C minimum air temperature) that analyzed ecosystem carbon fluxes associated with dominant shrub species *Cistus monspeliensis*. Our results show that modifications in plant morphology triggered by moderate warming enhanced plant photosynthesis, with no significant effect on ecosystem respiration, providing the first evidence for improved ecosystem carbon uptake in a dry shrubland under experimental warming.

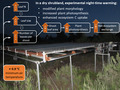

## 1 INTRODUCTION

The Earth's climate and carbon cycle are tightly coupled, such that changes in the physical climate (such as temperature increases) are likely to significantly affect carbon fluxes from terrestrial ecosystems, resulting in feedback patterns capable of accelerating climate warming (Ciais et al., 2013). The sensitivity of terrestrial CO_2_ fluxes to climate modification represents a large uncertainty for projections of greenhouse gas concentrations over the twenty‐first century (Higgins & Harte, 2012). For example, the response of terrestrial ecosystem carbon balances to climate change depends on how newly altered environmental conditions influence CO_2_ uptake and emissions. Rising temperatures can be expected to stimulate plant growth and ecosystem respiration (Rustad et al., 2001; Wu et al., 2011), but while one study suggested that this would also increase soil respiration rates (Bond‐Lamberty & Thomson, 2010), others suggested that such a temperature response may tend to disappear over time (Dieleman et al., 2012) and that the sensitivity of soil respiration to temperature may be unaffected by experimental warming across biomes (Carey et al., 2016).

The relative impact of climate change on such processes determines the direction and magnitude of carbon flux modifications. For example, in cold grassland areas, plant productivity is more stimulated than ecosystem respiration, leading to improved ecosystem carbon uptake (Wang et al., 2019), while the response of carbon fluxes to temperature increases can also vary by latitude and ecosystem type. To date, most warming studies have been conducted in high‐latitude or high‐elevation areas, with uncertain relevance to arid, semiarid, and dry subhumid ecosystems (Feng & Fu, 2013; Middleton & Thomas, 1997), where water rather than temperature constrains plant and soil processes (de Graaff et al., 2014). Although the relative importance of processes driving ecosystem responses to temperature increase can change in these regions, relevant warming experiments are rare. A coupled reduction of photosynthesis and soil respiration in response to warming, with no effect on net ecosystem exchange, was reported for a dryland plant community dominated by bunchgrasses (Wertin et al., 2017) and for a mixed‐grass prairie (Xu et al., 2016). In contrast, nighttime warming in a semiarid steppe was reported to improve net ecosystem exchange by suppressing soil respiration (Wang et al., 2020).

The climate change responses of some dryland ecosystem types, such as shrublands, have been especially neglected (Wu et al., 2011). Little information is available on the carbon flux dynamics of these ecosystems in response to climate warming, although shrublands represent 17.6% of global land cover (Hansen et al., 2000) and are a common ecosystem type in regions with high biodiversity, such as the Mediterranean basin (Mucina et al., 2017). Climate projections for this region indicate increasingly warmer conditions, especially during the summer season. By the end of the century, under the intermediate climate change scenario B1, temperature is expected to increase by 2°C in winter and 3°C in summer (Giorgi & Lionello, 2008). In the short term (2016–2035), under the similar intermediate scenario RCP 4.5, temperature is expected to increase by 0.5–1°C in winter and by 0.5–1.5°C in summer (Stocker et al., 2013). Climate manipulation experiments performed on these shrubland ecosystems have shown no (Darrouzet‐Nardi et al., 2018; de Dato et al., 2010; Emmett et al., 2004) or negative (García‐Palacios et al., 2018) warming impacts on soil CO_2_ fluxes, with no change in their sensitivity to temperature (Carey et al., 2016).

Similar experiments have also reported contrasting responses of plants to warming. Shoot length in the drought‐deciduous *Cistus monspeliensis* was not affected by warming (Penuelas et al., 2007), whereas significant growth and photosynthesis reduction were found in *Helianthemum squamatum* and other co‐occurring shrubs (León‐Sánchez et al., 2016, 2020). Reductions in leaf macro‐ and micronutrient concentrations (León‐Sánchez et al., 2020), and decreased nutrient resorption from senescent leaves (Prieto & Querejeta, 2020), have also been reported for these species. In *Erica multiflora*, experimental warming promoted shoot elongation (Prieto, Penuelas, et al., 2009), with no consistent effect on leaf photosynthesis (Liu et al., 2016). In contrast, an increase in photosynthesis rates was found in *Globularia alypum* (Prieto, Peñuelas, et al., 2009), which was associated with reduced shoot elongation (Prieto, Penuelas, et al., 2009). These inconsistent plant reactions to warming suggest that the processes leading to growth stimulation at higher latitudes and elevations, such as the release of temperature constraints on photosynthesis (Luo, 2007), the extension of the growing season (Forkel et al., 2016; Park et al., 2016), and accelerated organic matter mineralization (Bai et al., 2013; Grant, 2014), can have different influences on dry shrubland ecosystems. For example, in Mediterranean shrublands, where the temperature is already close to the optimal physiological range (Larcher, 2000), climate warming is expected to have little impact on plant growth (Way & Oren, 2010). Cold winter temperatures can restrain plant physiological processes in the Mediterranean climate (Flexas et al., 2014), and warming‐induced extension of the growing season has been reported for these ecosystems (Llorens et al., 2004; Prieto, Penuelas, et al., 2009).

Warming‐induced increases in evaporative water loss can result in dryer soils, limiting plant and soil processes (León‐Sánchez et al., 2016; Shaver et al., 2000). Soil dryness, which inhibits the diffusion of extracellular enzymes and soluble organic substrates to reaction microsites, represents an environmental constraint on organic matter decomposition (Davidson & Janssens, 2006). Under drier conditions, the warming‐induced acceleration of organic matter mineralization, usually observed in non‐water‐limited ecosystems, can be suppressed (Almagro et al., 2009) even if this effect is dependent on the plant species (Grossiord et al., 2018). Soil dryness can also reduce photosynthetic CO_2_ assimilation through diffusive and biochemical limitations to photosynthesis (Flexas et al., 2014; Limousin et al., 2010).

The effects of temperature increases on plants can be simulated by differing methods based on either active or passive ecosystem warming systems. The former (e.g., infrared lamps and heating cables) apply an external heat source to the ecosystem (Aronson & McNulty, 2009; LeCain et al., 2015), while the latter trap solar energy inside the ecosystem (e.g., field chambers for the greatest increase in maximum air temperature; Marion et al., 1997) or slow down heat loss from the ecosystem as air temperature decreases (e.g., nighttime warming for the greatest increase in minimum air temperature; Bruhn et al., 2013). Plant responses to experimental warming are influenced by the system adopted: Nighttime warming accelerates overnight carbohydrate depletion and can stimulate leaf photosynthesis the following day (Wang et al., 2020), while daytime warming can accelerate photosynthetic rates in cold climate (Peng et al., 2013; Sage & Kubien, 2007; Shaver et al., 2000) and increase the vapor pressure deficit (VPD) during the day. When high VPD is coupled with limited soil moisture, plants avoid hydraulic failure via stomatal closure, restricting photosynthetic activity (McDowell et al., 2008; Williams et al., 2013). These contrasting processes can explain the asymmetric responses of vegetation to increased maximum and minimum temperatures found in dry areas of the Northern Hemisphere (Peng et al., 2013; Tan et al., 2015).

We investigated changes in soil and plant processes determined by temperature increases in dryland ecosystems by carrying out a 10‐year (2001–2011) temperature manipulation experiment on a Mediterranean shrubland based on a nighttime warming system (Beier et al., 2004). During two consecutive growing seasons, we analyzed physiological and morphological changes in the dominant shrub species, their impact on plant carbon uptake capacity, and the consequent changes in ecosystem‐level carbon fluxes.

## 2 MATERIALS AND METHODS

### 2.1 Site description

The study area was located in Porto Conte Regional Park, northwestern Sardinia, Italy (40°37′48″N, 8°10′59″E). Climatic data (1971–2000) were collected from a weather station (Alghero Airport) 10 km away. The climate is dry subhumid, with a mean annual temperature of 16.8°C and a mean annual precipitation (P) of 573 mm. Rainfall mainly occurs from autumn to spring, with a long, dry period usually lasting from May to August. Potential evapotranspiration (PET) ranges from 951 to 1000 mm (data from Meteo‐Climatic Department of Sardinia Region) and the aridity index (P/PET) is 0.59 (Middleton & Thomas, 1997). The sandy loam soil is rocky and shallow (20–30 cm), with soil organic matter content of 3.9% in the main rooting zone (0–10 cm), total soil nitrogen content of 1.8 ± 0.2 mg g^−1^, soil mineral nitrogen (NO_3_^−^ + NH4^+^) content of 6.0 ± 0.9 µg g^−1^ (de Dato, 2003), and soil bulk density of 1.06 ± 0.11 g cm^−3^. The experimental site was covered by an open and scattered low shrubland community (garrigue) with a major incidence of drought‐deciduous species. The plot area consisted mainly of shrubs (82%) with a minor contribution from herbaceous species (7%). The semi‐deciduous shrub *Cistus monspeliensis* L. was the most abundant plant species, accounting for 32% of the area. See Figure S1 for further information on other species in the plant community.

### 2.2 Microclimate manipulation and monitoring

Microclimate manipulation was conducted from 2001 to 2011 (Beier et al., 2004; De Angelis et al., 2005). Both warming and control treatments were applied, with three replicate plots per treatment. Each plot was delimited by a metallic frame 6 × 4 m wide and 1 m tall. In the warming treatment, the metallic frame supported a mobile curtain made of aluminum strips knitted into a polyethylene mesh that reflected ~97% of the infrared (IR) radiation emitted by the soil at night. The curtains were moved by a motor automatically activated by an electronic controller. At sunset, when the light intensity dropped below 0.4 W m^−2^, the curtains were automatically drawn over the vegetation to reduce the loss of IR radiation. At sunrise, they were retracted to leave the plots open during the day. To avoid modifying the hydrological conditions, a sensor activated curtain removal when rain was detected.

In each plot, the following parameters were recorded on a half‐hour basis: air temperature at a height of 20 cm from the soil surface (Igromer HP100A, Rotronic, CH), soil temperature at 20 cm depth, and volumetric soil water content (WC) at 10 cm depth (ECH2O probe, Decagon Devices, Inc.). The WC values at saturation (usually in winter, December–February) ranged from 17% to 28%, while the minimum values (always in summer, July–August) ranged from 3% to 7%. Given the high heterogeneity in soil volumetric WC, we removed differences between plots by calculating the daily mean relative soil water content (RWC) as a percentage of the annual maximum, representing the value at saturation.

### 2.3 Shrubland CO_2_ exchange

Net ecosystem exchange (NEE, µmol CO_2_ m^−2^s^−1^) and total ecosystem respiration (TER, µmol CO_2_ m^−2^s^−1^) were measured 14 times from February 2010 to November 2011; gross photosynthesis (GP, µmol CO_2_ m^−2^s^−1^) was estimated by subtracting TER from NEE. Light use efficiency (LUE, µmol CO_2_ µmol photons^−1^) was calculated by dividing GP by the photosynthetic active radiation (PAR, µmol photons m^−2^s^−1^) recorded during the measurement. All measurements were performed at midday (between 11:00 and 13:00) under clear sky conditions.

Shrubland CO_2_ exchange was measured using transparent canopy chambers fitted with square NEE soil collars (0.64 m^2^) connected to an LI‐8100 infrared gas analyzer (LI‐COR Biosciences, Inc.), following Guidolotti et al. (2017). Two NEE collars were installed in each plot (six per treatment) during winter 2009, after 8 years of continuous temperature manipulation. The heterogeneity of the plant community and the limited size of the canopy chambers meant that the NEE collars could not include a fully representative patch (by structure and composition) of the garrigue community. Therefore, we selected portions of garrigue where *Cistus monspeliensis* (and secondarily *Cistus creticus*) were dominant. *C. monspeliensis* is the most abundant species in the plant community and is characterized by higher plant‐level photosynthetic rates than co‐occurring species (Liberati et al., 2018). One to three *Cistus* specimens were included in each NEE collar (Table S1). In seven of 12 collars, a *C. monspeliensis* specimen was also monitored for leaf physiology, morphology, and chemistry. Images of the vegetation included in the NEE collars were taken four times in 2010. An object‐oriented classification method using the segmentation algorithm embedded in eCognition Developer 9.5 (Trimble Germany GmbH) was applied to these images to assess the percentage of the NEE collar area occupied by the different species (Chenari et al., 2017).

Soil respiration (SR, µmol CO_2_ m^−2^s^−1^) was measured in two round PVC collars (10 cm diameter) placed within each 0.64 m^2^ collar using an LI‐8100 equipped with an 8100‐102 soil chamber (10 cm). A previous study carried out at this experimental site compared the warming effect on SR measured at sunrise (just after the nighttime warming period) and midday (de Dato et al., 2010). As the warming effect on SR was similar (no effect) at these two times, we decided to measure SR at midday, together with the other ecosystem CO_2_ fluxes.

### 2.4 Leaf gas exchange and water potential

Leaf gas exchange and water potential measurements were repeated 11 times from March 2010 to November 2011 on the same 12 *C. monspeliensis* specimens (two specimens per plot, six per treatment), seven of which were also included in the NEE collars. The predawn leaf water potential (ψpd, MPa) was measured using a Scholander pressure chamber. Photosynthesis (*A*, µmol CO_2_ m^−2^ s^−1^) and stomatal conductance (*g*_s_ mol H_2_O m^−2^ s^−1^) were measured in situ at midday (between 11:00 and 13:00) using an LI‐6400 portable photosynthesis system (LI‐COR Biosciences, Inc.) equipped with a conifer chamber (6400‐22 Opaque Conifer Chamber, in combination with 6400‐18 RGB light source). The microclimate inside the leaf chamber (CO_2_ concentration, temperature, and light intensity) was set according to the actual conditions occurring under clear sky conditions at the beginning of each measurement campaign, which usually lasted for 3 days.

Due to the small size and the very short petiole of *C. monspeliensis* leaves, it was not possible to measure these physiological parameters in single leaves, so we included an entire shoot in the leaf chamber of the gas exchange analyzer and the pressure chamber. Shoots with morphology such as those labeled for shoot development monitoring (see following paragraphs) were selected each time for gas exchange and water potential measurement.

The apparent maximum rate of carboxylation by Rubisco (*V*_cmax_) and the maximum rate of electron transport (*J*_max_) were calculated from the photosynthesis‐CO_2_ response curves (*A*–*C*_i_ curves; Long & Bernacchi, 2003), according to the Farquhar model of leaf photosynthesis. Shoots were collected the day before the execution of the *A*–*C*_i_ curves; cutting was performed under water to prevent xylem cavitation. The shoots were kept with their bases submerged in water overnight until measurement to ensure full hydration of the leaves and therefore adequate stomatal conductance (necessary to obtain a correct *V*_cmax_ and *J*_max_ estimate) in the dry season. Inside the leaf chamber, a temperature of 25°C and quantum flux density of 1500 µmol photons m^−2^ s^−1^ were set. After gas exchange measurements (*A*–*g*_s_ and *V*_cmax_–*J*_max_), the leaves of the shoot were detached, and the total shoot leaf area was recorded with a scanner, avoiding the shrinkage of leaves caused by desiccation.

### 2.5 Leaf morphology and nitrogen content

Leaf morphology and chemistry were monitored 15 times from July 2009 to November 2011. Eighteen *C. monspeliensis* specimens (three per plot, nine per treatment), including all specimens selected for leaf gas exchange measurement and shoot development monitoring (see following paragraph), were identified at the beginning of the study period and repeatedly sampled. During each sampling, a shoot was collected from each monitored specimen, and an image of all leaves composing the shoot was acquired using a scanner. The image was then analyzed to measure the area and length/width ratio of each leaf. The leaf mass per area (LMA, g m^−2^) was determined after drying the leaves at 70°C until the weight was constant. Leaf samples were then ground and the nitrogen content (N, expressed by both leaf area and mass) was determined using an elemental analyzer (Carlo Erba model 1108EA). The ratio between A (µmol CO_2_ m^−2^ s^−1^) and leaf N content expressed on a leaf area basis (g m^−2^) was used to calculate photosynthetic nitrogen use efficiency (PNUE, µmol CO_2_ gN^−1^ s^−1^).

### 2.6 Shoot development

The growth of *C. monspeliensis* shoots was monitored during two consecutive growing seasons, from July 2009 to June 2011. The beginning of the growing season was defined as the occurrence of vegetative stasis during summer drought. During this period, most leaves were shed, and the canopy was only composed of short new shoots formed in spring (brachyblasts). When the first autumn rains increased the soil WC, the brachyblasts began to produce new leaves and elongate, producing long shoots (dolichoblasts). During winter and spring, dolichoblast growth continued, forming new brachyblasts in the leaf axils. Based on this development pattern (de Micco & Aronne, 2009), in July 2009 and July 2010, five brachyblasts were labeled on three specimens per plot (subjected to the physiological and morphological measurements described above). These shoots were monitored approximately every 2 months until the next summer, recording the shoot length, number and type of leaves, and length of each leaf. We calculated the width of each leaf of the labeled shoots based on the leaf length/width ratio, determined by the leaves collected and scanned after gas exchange measurements carried out on the same sampling date, then estimated the leaf area by approximating the leaf shape to an ellipse. The shoot leaf area was obtained by summing the leaf area of all leaves recorded on the shoots.

### 2.7 Leaf litter production and nitrogen resorption efficiency

Two litter traps (157 cm^2^ aperture) were placed below each *C. monspeliensis* specimen selected to monitor shoot development. Litter was collected at approximately 2‐month intervals during two full years (September 2009 to September 2011). After collection, the litter was dried at 70°C to a constant weight, then sorted into leaves, branches, and reproductive parts before weighing. Leaf litter nitrogen content (indicating N resorption proficiency) was determined as described above for leaves. N resorption efficiency from senescent leaves was calculated following Killingbeck (1996): (Leaf N − leaf litter N)/leaf N*100, where leaf N and leaf litter N are the average N content (%) of leaf and leaf litter over the entire study period.

### 2.8 Statistics

Repeated ANOVA was applied to estimate the treatment effect over the entire study period (Filella et al., 2004; Lellei‐Kovács et al., 2008). On each sampling date, the warming effect was evaluated using the Fisher LSD post hoc test. The sample units for statistical analyses were the plot for microclimate data (*n* = 3) and the single *Cistus* specimen for plant data (*n* = 6 for leaf and ecosystem gas exchange parameters, *n* = 9 for shoot development, litterfall, and leaf chemistry parameters). Absolute and relative warming effects were calculated as W–C and (W–C)/C × 100, respectively. Unpaired two‐tailed *t* tests were used to test the differences in resorption efficiency. All analyses were performed using GraphPad Prism (version 9.0.0; GraphPad Software).

## 3 RESULTS

### 3.1 Treatment effect

**FIGURE 1:**
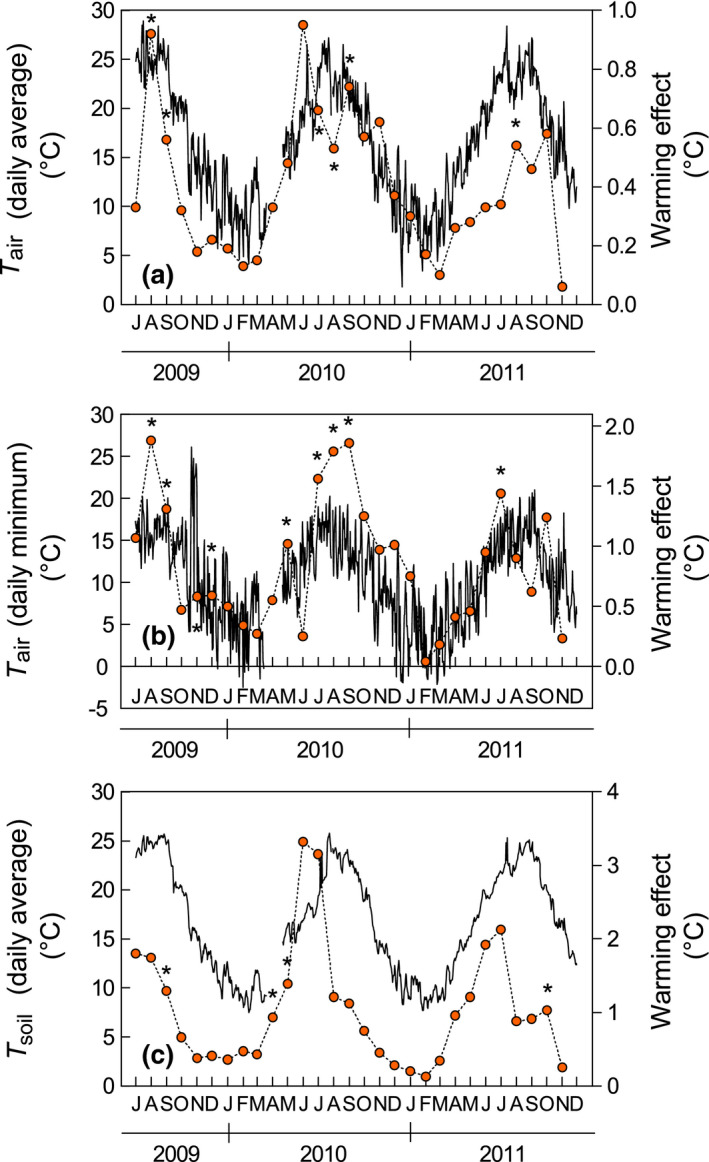
Seasonal trends in (a) daily average air temperature, (b) daily minimum temperature, and (c) daily average soil temperature at 20 cm depth measured in control plots (solid line). Right‐hand *y* axis shows the monthly average of the warming‐control difference (dashed line). *indicates significant (*p* < 0.05) warming effect for a single month

The nighttime warming treatment significantly modified the plots’ microclimate, increasing the daily mean air temperature by 0.41°C (*p* = 0.06, Figure 1a) and the daily minimum air temperature by 0.87°C (*p* < 0.01, Figure 1b) but not significantly affecting the daily maximum temperature (*p* = 0.71). Daily mean soil temperature averaged 1.04°C higher under the treatment (*p* = 0.02, Figure 1c). The magnitude of changes in air and soil temperature was maximized in summer, but RWC was not significantly affected by the treatment (*p* = 0.15; Figure S3). The number of days with minimum air temperature below 0°C was 30 in the control plots and 20 in the warming plots.

### 3.2 Ecosystem CO_2_ exchange dynamics

The characteristics of the plant community patches monitored for ecosystem gas exchange are shown in Figure S2. Inside the NEE collars, the highest contribution to the plant cover was found in *Cistus monspeliensis*, with minor contributions from other shrub species, such as *Cistus creticus*, *Dorycnium pentaphyllum*, *Daphne gnidium*, *Helichrysum microphyllum*, and *Rosmarinus officinalis*. In the warming collars, where plants experienced a long (8–10 years) acclimation to the manipulated environmental conditions, the vegetation was more developed, with a higher degree of plant and *Cistus* spp. cover (i.e., more expanded canopies with fewer gaps).

**FIGURE 2:**
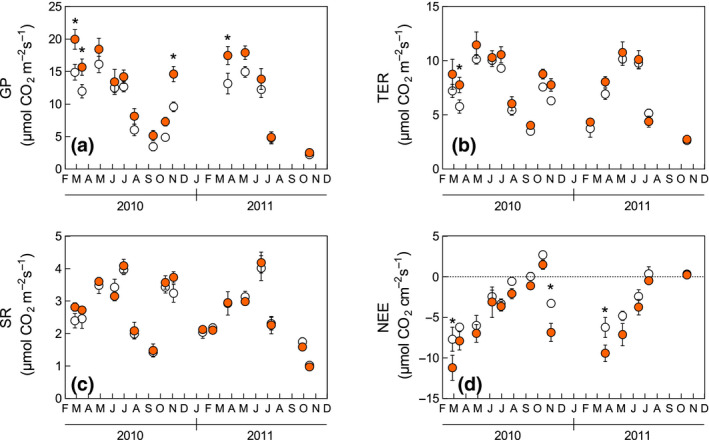
(a) Gross photosynthesis, (b) total ecosystem respiration, (c) soil respiration, and (d) net ecosystem exchange during the study period. White and shaded circles indicate control and warming treatments, respectively. Data represent means ± standard error (*n* = 6). *indicates a significant (*p* < 0.05) warming effect on a single measurement date

The CO_2_ fluxes measured at the ecosystem level showed significant seasonal variability, which was repeated in both years of measurement. Over the study period, the warming treatment increased GP (+29.5%, *p* = 0.03, Figure 2a), with the most significant changes recorded when plant activity was not limited by soil water availability. In contrast, no significant differences between treatments were recorded for TER and SR (Figure 2b,c). As a result, NEE was greater (more negative) in the warming plots (+46.8%, *p* = 0.03, Figure 2d). Light‐use efficiency was also higher in the warming treatment (0.0080 ± 0.0010 µmol CO_2_ µmol photons^−1^ in control vs. 0.0098 ± 0.0014 µmol CO_2_ µmol photons^−1^ in warming; warming effect = +24%, *p* = 0.05).

### 3.3 Leaf physiology

**FIGURE 3:**
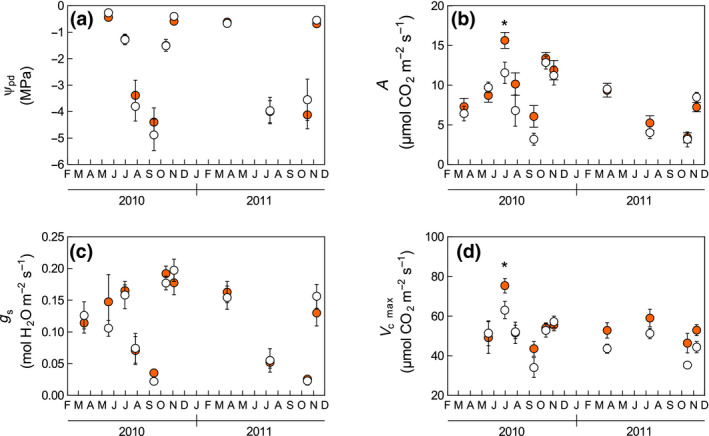
(a) Predawn leaf water potential, (b) leaf photosynthesis, (c) stomatal conductance, and (d) apparent maximum rate of carboxylation by RuBisCO (*V*_cmax_). White and shaded circles indicate control and warming treatments, respectively. Data represent means ± standard error (*n* = 6). *indicates a significant (*p* < 0.05) warming effect on a single measurement date

Based on fluctuations in RWC (Figure S3), *ψ* showed a clear seasonal trend, with the lowest values occurring during summer (Figure 3a). During the dry period, low water availability also constrained A (Figure 3b), *g*_s_ (Figure 3c), and *V*_cmax_ (Figure 3d). Over the full study period, *ψ*, *g*_s_, and PNUE (Figure S4a) were not significantly modified by the warming treatment. Warming increased A in June 2010, but considering the entire study period, the treatment effect was scarcely significant (*p* = 0.09). In contrast, warming increased *V*_cmax_ by 10% (*p* = 0.01) and *J*_max_ by 7% (*p* = 0.02, Figure S4b). Photosynthesis measured concurrently with *V*_cmax_ estimation on detached hydrated shoots was also increased by warming (+7%, *p* = 0.06, Figure S4c).

### 3.4 Shoot development and leaf litter production

**FIGURE 4:**
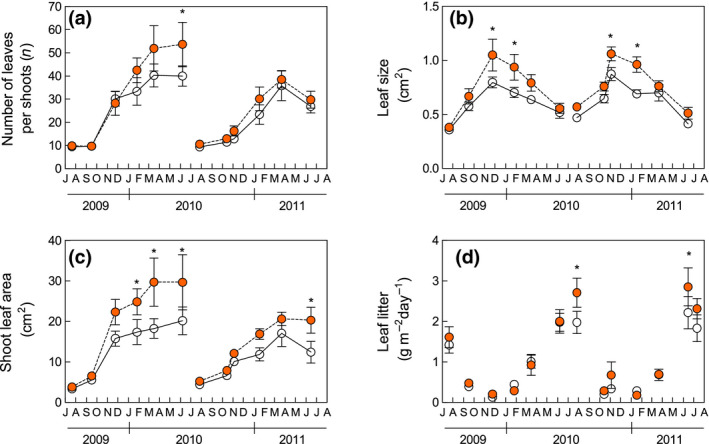
(a) Number of the leaves per shoot, (b) leaf size, (c) total shoot leaf area, and (d) litter production during two growing seasons (each value represents an average for the previous time interval). Lines connect values belonging to the same shoot cohort. White and shaded circles indicate the control and warming treatments, respectively. Data represent means ± standard error (*n* = 9)

In June 2010, once shoot development was complete and before the beginning of leaf shedding, the number of leaves per shoot had increased by 34% over control (*p* = 0.04, Figure 4a), though this effect was not significant during the subsequent growing season. Throughout the study period, leaf size was significantly larger under the warming treatment (+21.8%, *p* = 0.01, Figure 4b). The increase in leaf number and leaf enlargement resulted in a wider total shoot leaf area in warmed plots (+38.4%, *p* = 0.02, Figure 4c), whereas shoot length was not modified (Figure S5). Litter was mainly produced in June and July (Figure 4d) when most leaves were shed to face the summer dry period. At the seasonal peak of leaf shedding, the mass of *C. monspeliensis* leaf litter produced in warming plots was significantly higher than that in the control (+37% in July 2010, *p* = 0.02; +28.5% in June 2011, *p* = 0.04). Leaf litter production over the entire study period (July 2009 to October 2011) was also significantly higher in the warming plots (+15% in October 2011, *p* = 0.04, Figure S6).

### 3.5 LMA, leaf N content, and N resorption efficiency

**FIGURE 5:**
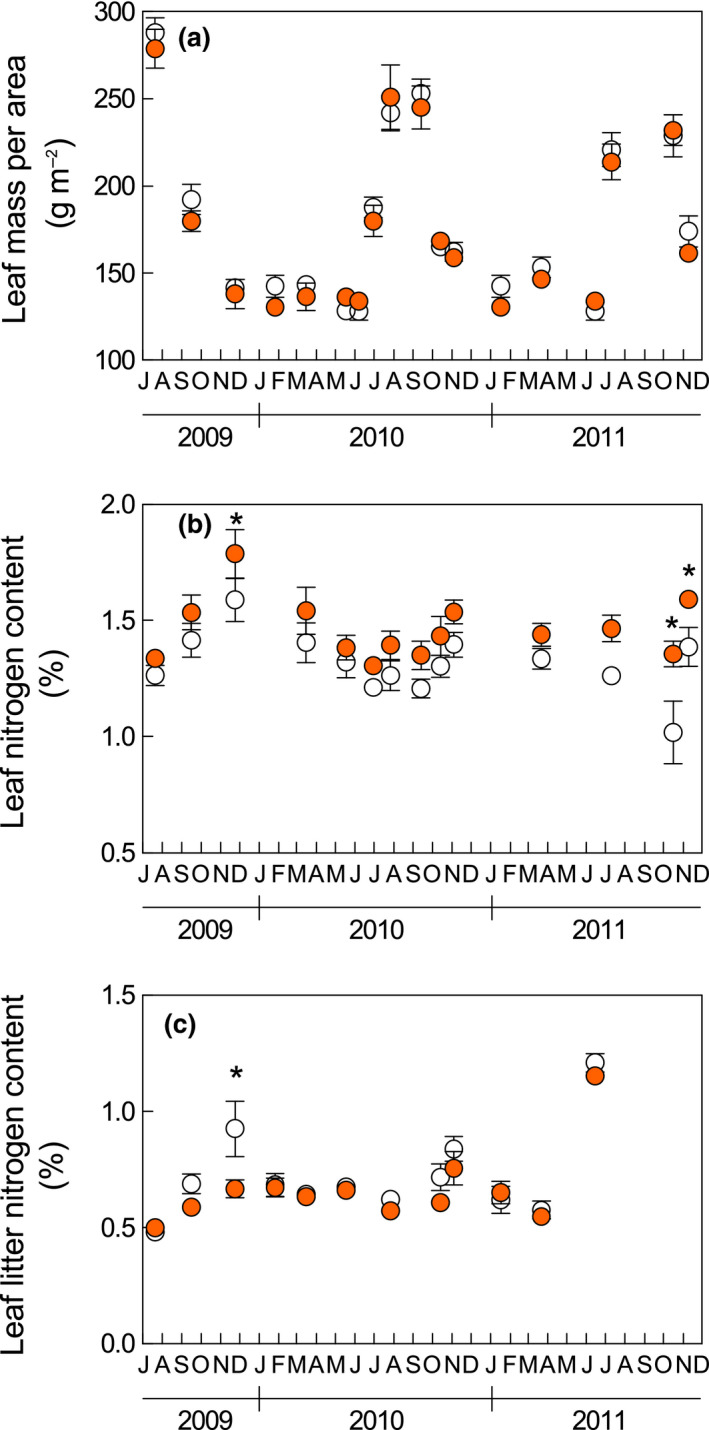
(a) Leaf mass per area, (b) leaf nitrogen content, and (c) leaf litter nitrogen content. Data in (a) and (b) represent the average shoot value (including brachyblasts and leaves). White and shaded circles indicate the control and warming treatments, respectively. Data represent means ± standard error (*n* = 9). *indicates significant differences for a single measurement date

**FIGURE 6:**
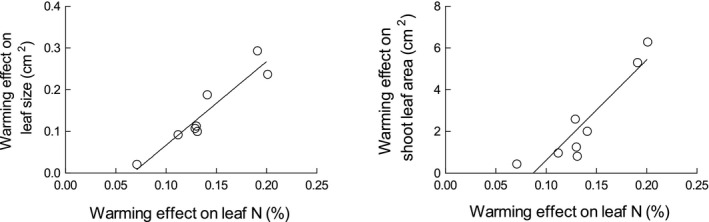
Relationship between the warming effect on leaf N content and (a) warming effect on leaf size (*p* < 0.01) and (b) shoot leaf area (*p* < 0.01), excluding brachyblast leaves. Each point represents the warming effect on one sampling date

Due to the different leaf typologies present on the shoots during the year, the average LMA of the shoot leaves showed significant seasonal fluctuations (Figure 5a), reaching the maximum value during the summer dry period, when summer leaves were the prevalent leaf typology. Leaf N also changed according to these different leaf typologies (Figure 5b), reaching its lowest values in the summer. The warming treatment did not affect either LMA or carbon content (expressed both on leaf area and mass basis, Figure S7), but increased leaf nitrogen content (average warming effect +11%, *p* < 0.01). Conversely, leaf litter nitrogen concentration (an estimate of leaf N resorption proficiency) was slightly reduced by warming (− 6%, *p* = 0.07). As a result of both high N and low N leaf litter, N resorption efficiency was higher in the warming treatment (73.8% vs. 69.1% in control, *p* <0 .01). The warming effect on both leaf size and shoot leaf area was positively related to the warming effect on leaf nitrogen content (Figure 6a,b).

## 4 DISCUSSION

The nighttime passive warming system produced a moderate increase in minimum air temperature, with an effect on mean daily temperature in the lower range foreseen in the short term (2016–2035) for the Sardinia region (Stocker et al., 2013). As this area is expected to experience greater climate warming (+2–3°C) by the end of the century (Giorgi & Lionello, 2008), the results of this experiment are relevant to understanding the current and forthcoming ecosystem responses expected under intermediate scenarios. Even if both maximum and minimum temperatures are expected to increase (Stocker et al., 2013), the greater increase in minimum temperature obtained in this experiment mirrors the reduction in diurnal temperature range (greater increase in minimum than in maximum temperatures) observed from the second half of the previous century (Donat & Alexander, 2012).

The seasonal and daily distribution of temperature changes is an important factor controlling the dominance of ecosystem processes involved in plant and soil responses (Peng et al., 2013; Tan et al., 2015; Xia et al., 2009, 2014). In a dry ecosystem, daytime warming can exacerbate water limitations on plant physiological activity through enhanced evaporation and increased vapor pressure deficit (León‐Sánchez et al., 2016; Peng et al., 2013). In contrast, in our experiment, the maximum warming effect on temperature was produced at night, such that no direct interference with photosynthetic activity occurred during the day. Similar to other passive nighttime warming experiments (Lellei‐Kovács et al., 2008; Llorens et al., 2004), we did not observe significant soil drying. Reduced air humidity and dew formation were observed in the warming plots at night (Bruhn et al., 2013). As already noted by previous research (Amthor et al., 2010; Beier et al., 2004; Bruhn et al., 2013), this effect represents an artifact introduced by experimental temperature manipulation. Dewfall represents an important source of water for ecosystems in arid and semiarid areas (Uclés et al., 2014), especially for biocrust formation (Baldauf et al., 2021). In our experiment, however, the predawn leaf water potential of *C. monspeliensis* was not modified by warming, even during dry periods, indicating that both nighttime soil warming and reduced dew formation did not significantly modify plant water balance.

Under these slightly modified climatic conditions, we observed significant variations in ecosystem carbon fluxes, with more negative NEE values (i.e., enhanced ecosystem‐level carbon uptake) observed under warming treatment. With no significant effect recorded in SR, and a limited (not significant) increase in TER, NEE stimulation mainly depended on the higher GP of vegetation in the NEE collars. Gross photosynthesis displayed strong seasonality, being lowest during the summer dry period and highest in spring and autumn (Guidolotti et al., 2017). The warming effect was restricted to these periods of optimal environmental conditions, and therefore did not depend on a different response to the summer aridity triggered by the temperature increase. Moreover, ecosystemic fluxes were affected by the structure and composition of vegetation in the canopy chamber. The total plant cover in the NEE collars as well as the *Cistus‐specific* cover were higher under warming conditions, while the TER measured in the two treatments indicated a comparable living biomass. Hence, a higher GP reflected an increased light‐use efficiency of the plants included in the warming collars, suggesting structural changes in the photosynthetic capacity of the plant cover.

GP depends on the complex interactions of single‐leaf photosynthesis, canopy leaf area, and canopy architecture (Muraoka et al., 2010). We found a moderate positive effect of warming on leaf photosynthesis values measured in detached, hydrated shoots. When measured in the field at midday, however, leaf photosynthesis was similar in the warming and control plots for most of the study period. Given that *g*_s_ measured in the field was, on average, half of *g*_s_ measured on hydrated shoots, this stomatal closure likely restricted the higher photosynthetic capacity (attested by the higher *V*_cmax_) of warming shoots under field conditions. Therefore, leaf‐level photosynthesis enhancement was not the main factor responsible for the improved GP measured in the field at midday, and other factors should be considered. In particular, the increased leaf litter production suggested a change in plant morphology, with denser canopies (higher leaf area index) formed in the warming plots. The leaf area index is the major determinant of gross primary production (Poyatos et al., 2014), indicating that the higher leafiness of warmed plants was one of the main factors underlying the observed NEE improvement. This change in canopy structure reflects morphological modifications occurring at the shoot level. Even if shoot length was not affected (De Dato et al., 2008; Penuelas et al., 2007), plants subjected to nighttime warming showed a wider shoot leaf area than control plants. This difference depends on two modifications: (1) an increase in leaf size and (2) an increase in the number of leaves produced during the season (Figure 4).

Similar shoot growth stimulation has been frequently observed under experimental warming and can depend on different mechanisms, such as an increase in photosynthetic rates related to the kinetic sensitivity of photosynthesis to temperature (Luo, 2007), stimulation of photosynthesis by the enhanced consumption of carbohydrates during warmer nights (Turnbull et al., 2004; Wan et al., 2009), lengthening of the growing season (Forkel et al., 2016; Park et al., 2016), and acceleration of nitrogen mineralization, which improves soil nutrient availability (Grant, 2014).

A direct temperature impact on photosynthesis kinetics was excluded in the present experiment based on nighttime warming: The nighttime increments of air temperatures were gradually lost after curtain removal, and no significant differences in maximum air temperature were found. Furthermore, the limited effect of warming on leaf photosynthesis measured in the field suggests only a minor role for nighttime carbohydrate depletion in stimulating midday leaf carbon uptake. Growing season elongation in response to increased temperature is more relevant in colder climates, but has also been reported for similar nighttime warming experiments carried out in a Mediterranean climate (Llorens et al., 2004; Prieto, Penuelas, et al., 2009). The flushing of new *C. monspeliensis* leaves mostly occurred from October to April (Figure 4), and we cannot exclude the positive impact of higher winter temperatures on this process.

Despite the possible impact of growing season elongation, nitrogen mineralization stimulation appeared to be the main process involved in the observed plant growth stimulation. This hypothesis is supported by the higher N mineralization rates previously found in warming plots during the cold season (de Dato, 2003) and by the higher leaf N content of warmed plants observed in this study (Figure 5b). Improved N mineralization was consistently found under experimental warming in a range of ecosystem types (Bai et al., 2013), which can depend on several processes (Zhang et al., 2016). First, higher soil temperature can directly stimulate organic matter decomposition affecting (1) the activity of the extracellular enzymes responsible for the depolymerization of soil organic matter and (2) the non‐covalent interactions that bind organic matter to mineral particles in soil aggregates (Conant et al., 2011). Second, soil warming can indirectly modify the size and composition of the microbial community, but unfortunately no generalized response patterns can be determined as this has been reported to have positive, negative, and neutral effects (DeAngelis et al., 2015; Hu et al., 2016). Third, experimental warming may stimulate organic matter decomposition and soil N cycling by increasing the input of labile carbon through root exudation (Yin et al., 2013; Zhang et al., 2016), which fuels N microbial transformations (Paterson, 2003).

The microbial biomass response to warming was investigated at the experimental site in two different studies carried out in January 2012 and October 2012. The first (sampling depth 0–5 cm) did not identify a significant effect of warming on microbial biomass (Rousk et al., 2013), while the second (sampling depth 0–10 cm) found 20% more microbial biomass in the warming plots and similar amounts of extractable carbon (a proxy of root exudates) between the two treatments (Gavrichkova et al., 2017). These results indicate a minor role for root exudation, suggesting that, along with the direct temperature stimulation of organic matter decomposition, the growth of microbial communities in warmed plots may also have contributed to accelerating the nutrient mineralization rates.

Most soil N is in organic form, with mineral N directly available for plants representing ~1% of the total N pool (Bingham & Cotrufo, 2016; Sylvia et al., 2005; Wen et al., 2016), so N mineralization is a key process controlling net primary production (Schimel et al., 1997). Mineral N at our site represented 0.3% of the total soil N, suggesting a significant restriction of N mineralization on the amount of N available for plant growth. Moreover, plant growth across biomes is N‐limited (LeBauer & Treseder, 2008), and positive plant productivity responses to experimental N input have also been found in dryland ecosystems (Hooper & Johnson, 1999). In addition, experimental warming combined with enhanced drought was found to ameliorate the nutritional state of tree species in dry woodlands and buffer the additional water stress imposed by rainfall exclusion (Grossiord et al., 2018).

The improved organic matter mineralization found at our site may have increased the soil nutrient availability, determining the redistribution of N from soil to vegetation and explaining the increase in leaf and shoot size observed in the warming treatment (Figure 4). A similar increase in shoot size has been reported for shrubs subjected to increasing N availability, as a consequence of both direct N addition (Bowman & Conant, 1994; Muñoz et al., 2005) and experimental soil warming (Koller et al., 2016). Similarly, higher N availability can be responsible for positive feedbacks on leaf size, which depends on the number (related to cell replication) and the size (related to cell expansion) of cells formed during leaf ontogeny. Cell replication and expansion are under genetic and environmental constraints, and the latter determines the phenotypic plasticity of leaf size (Van Volkenburgh, 1999). Among the environmental factors responsible for the intraspecific variability of this trait, soil fertility is positively related to leaf size (Gray & Schlesinger, 1983; McDonald et al., 2003; Meier & Leuschner, 2008). In particular, there is evidence that an increased NO_3_‐supply in the soil can modulate leaf expansion through a cytokinin signal exported from root to shoot, where leaf cell division is promoted (Van der Werf & Nagel, 1996; Walch‐Liu et al., 2005). A similar N‐induced stimulation of cell division may have also occurred in *C. monspeliensis* plants subjected to warming. The leaf lamina became wider, without changes in the macroscopic anatomical properties of the leaf (no differences in LMA, Figure 5b). A similar LMA excludes the enhancement of cell expansion and supports the hypothesis of stimulation of cell replication. In support of the role played by higher N availability in modifying shoot morphology, both leaf size and shoot leaf area were positively related to leaf N content, with differences in the latter between warmed and control plants explaining 70% of the leaf size variability and 55% of the shoot leaf area variability (Figure 6).

In addition to the stimulation of soil N mineralization, another process may have contributed to ameliorating plant nutrient status in the warming treatment. The amount of nutrients available for plant growth also depends on nutrient recovery from senescent organs. Both N resorption proficiency and efficiency indicated better N resorption from senescent leaves under warming conditions. This warming effect was possibly related to the redistribution of N from cell walls, hardly translocated during senescence (Cárdenas & Campo, 2007), to the photosynthetic apparatus. The amount of N in the cell wall matrix can increase in response to chilling temperatures due to adjustments in cell wall rigidity (Scholz et al., 2012; Solecka et al., 2008), and this has been associated with reduced N resorption efficiency along temperature gradients (Cong et al., 2019), even in Mediterranean ecosystems (González‐Zurdo et al., 2015). A similar temperature response could be responsible for the higher resorption proficiency (lower N concentration in the leaf litter) observed during warming. Unlike *Cistus* plants subjected to short‐time warming experiments (De Micco et al., 2011; Vitale et al., 2014), in this study, LMA and leaf carbon content did not change with warming. Hence, we can exclude the notion that the lower number of cold nights experienced by the warmed plants reduced the cell wall thickness. In contrast, we hypothesize a reduction in proteins with a frost protection function associated with the cell wall, with a redistribution of the N leaf pool toward photosynthetic proteins. This is consistent with the higher *V*_cmax_ measured in warming plants. Interestingly, a reduced N resorption efficiency was found in a dry shrubland subjected to +2–4°C warming, possibly related to an inhibition of physiological processes involved in resorption, as well as a lower N content found in mature leaves (Prieto & Querejeta, 2020).

In conclusion, the release of temperature limitation on N mineralization and a better N recovery from senescent leaves caused N redistribution from soil organic matter to vegetation, improved plants’ nutritional state, and led to a change in shoot and canopy morphology. The formation of denser canopies in the warming plots agreed with the higher *Cistus* cover in the NEE collars and with greater GP rates. To the best of our knowledge, such GP and NEE enhancement under increased temperature has not been previously reported for dry shrublands. In contrast, similar warming experiments carried out in semiarid climates found reduced soil water availability, restricted nutrient cycling, worsened the plants’ nutritional state, and constrained the photosynthetic capacity of the shrub *Helianthemum squamatum* (*Cistaceae*; León‐Sánchez et al., 2016, 2018; Prieto & Querejeta, 2020). These contrasting responses for similar species may depend on differences in warming type (passive nighttime warming vs. open‐top chamber), warming intensity (+0.4°C vs. +2°C), and climate (dry subhumid vs. semiarid) between these experiments. In our study, nighttime warming did not increase VPD during the day when plant photosynthesis occurred, and the lower warming intensity did not significantly modify the soil moisture and the plant water status. Differently, in León‐Sánchez et al. (2016, 2018), a drier climate exacerbated the detrimental effects of the water limitation due to warming on plant physiological activity. In our study, the mildness of the warming treatment applied and the relatively mesic climate of the area may have been responsible for the positive plant responses observed. Finally, without an increase in PNUE or additional N input, progressive N sequestration in plant biomass could progressively reduce soil N availability, exacerbating N limitations in the warmed ecosystem and possibly offsetting this plant response to warming (McKinley & Blair, 2008). This beneficial outcome of a slight change in temperature was observed after 10 years of climate manipulation, indicating that in the medium term, the soil N pool can sustain an enhanced ecosystem carbon uptake.

## CONFLICT OF INTEREST

There are no conflict of interest to report.

## Supporting information

Supplementary Material

## Data Availability

The data that support the findings of this study are openly available in Figshare at https://figshare.com/s/bde5790b17ac4cd1689c.
