# Supplementary material for: Enhancement of ecosystem carbon uptake in a dry shrubland under moderate warming: The role of nitrogen‐driven changes in plant morphology
Source: Glob Chang Biol. 2021 Aug 16;27(21):5629–42. doi: 10.1111/gcb.15823 (PMC9290483; doi:10.1111/gcb.15823)
Supplement: Supplementary file 1 — Supplementary Material [file GCB-27-5629-s001.docx]

Table S1 Characteristics of the plant community patches included in the NEE collars. Data represent means ± standard error (n=6). Different letters represent significant differences between Control and Warming

|  | Control | Warming |
| --- | --- | --- |
| Cistus specimens (n) | 1.5 ± 0.2 a | 1.3 ± 0.3 a |
| Total plant cover degree ( % of the collar area) | 76 ± 1 % a | 85 ± 1 % b |
| *Cistus* spp. cover degree (% of plant cover) | 67 ± 5 % a | 95 ± 1 % b |

Figure S1. Relative cover degree of the shrub and herbaceous species in control and warming plots in June 2010. Data represent means ± standard error (n=3). Relative cover degree (also including dead plants parts) was obtained through the pinpoint method, using the methods described in (Liberati et al., 2018).

Figure S2 Species plant cover inside the NEE collars during 2010 in control and warming plots. Data represent means ± standard error (n=6).


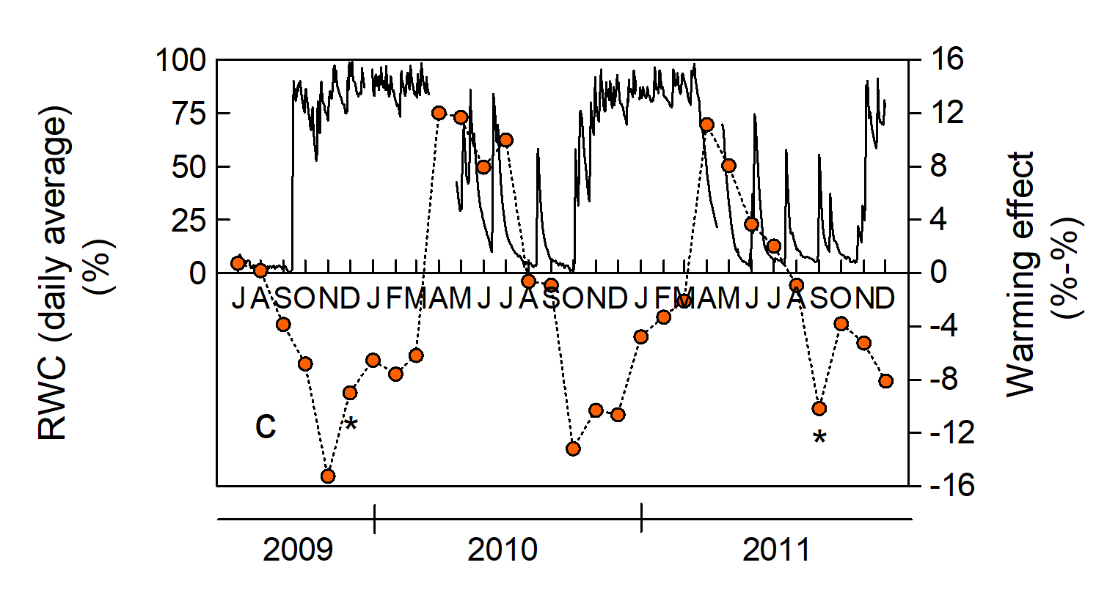


Figure S3 Seasonal trend of Relative soil content (RWC) in Control plots (solid line). On right Y axis is reported seasonal trend of the Warming effect on RWC (monthly average of the Warming-Control differences (broken line and closed circles). * indicates significant (p<0.05) differences on a single month.


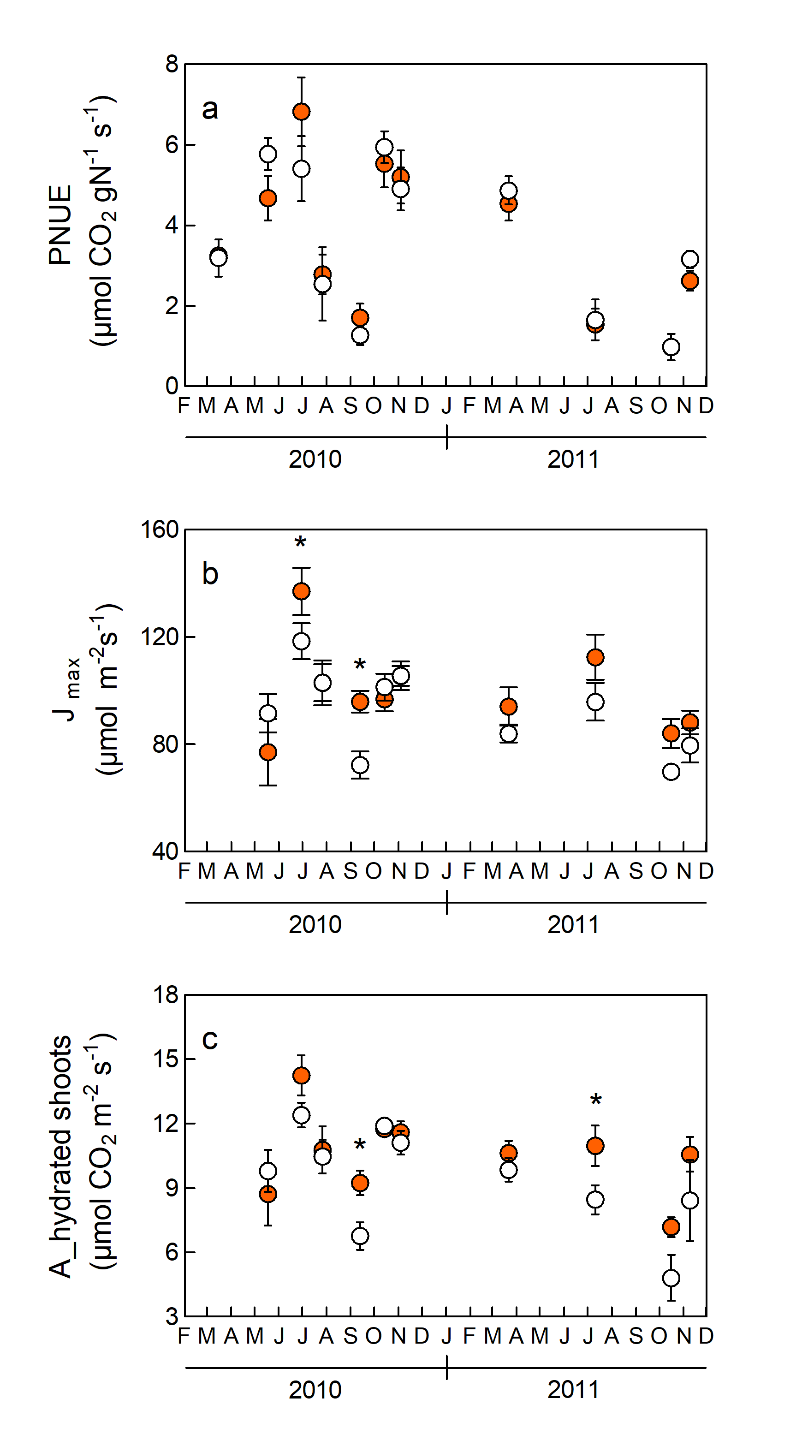


Figure S4 Seasonal trend of the PNUE (a), J_max_ (b) and A measured on detached, hydrated shoots (c). Open circles indicate Control, closed circles Warming. Data represent means ± standard error (n=6). * indicates significant (p<0.05) differences on a single date


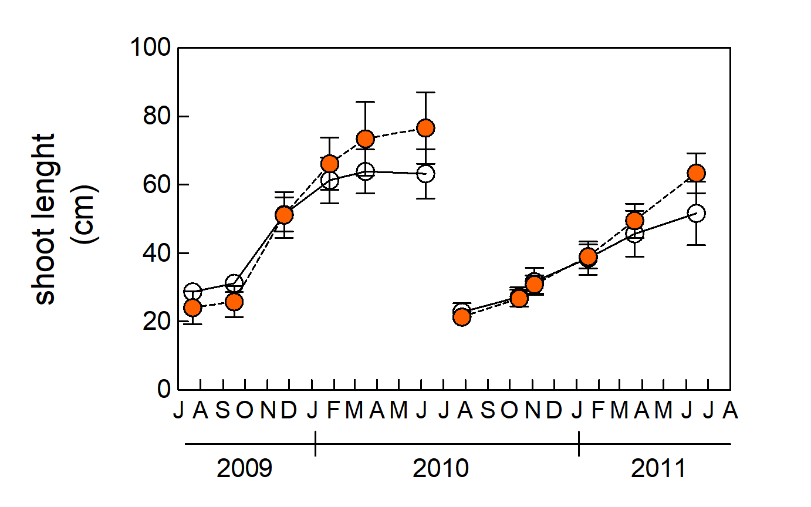


Figure S5 Seasonal trend of the shoot length. Open circles indicate Control, closed circles Warming. Data represent means ± standard error (n=9). * indicates significant (p<0.05) differences on a single date


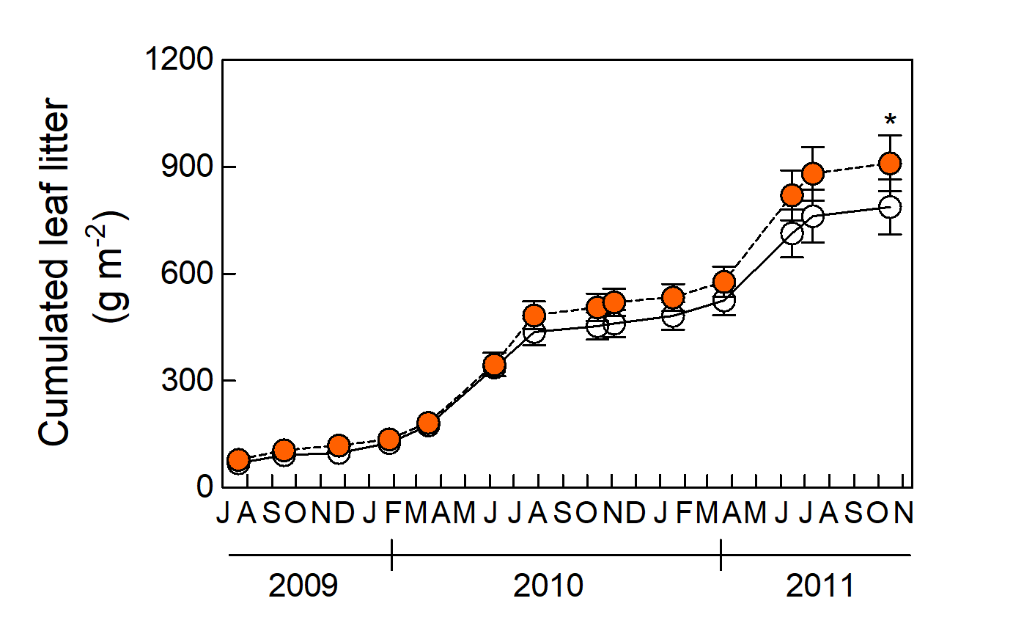


Figure S6 Cumulate leaf litter production over the whole study period. Open circles indicate Control, closed circles Warming. Data represent means ± standard error (n=9). * indicates significant (p<0.05) differences on a single date


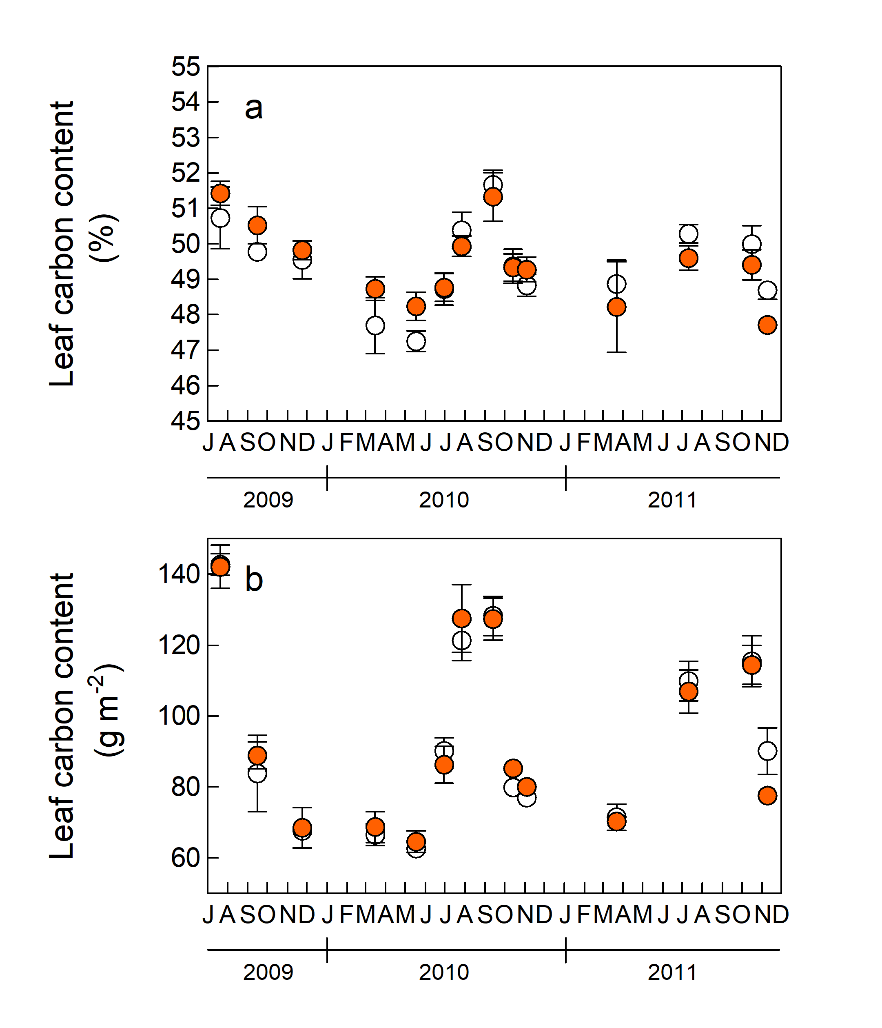


Figure S7 Leaf carbon content on leaf mass (a) and leaf area basis over the study period. Open circles indicate Control, closed circles Warming. Data represent means ± standard error (n=9)
